# Comparative Efficacy of AZD9496 and Fulvestrant on the Growth of Pituitary Adenoma via Blocking JAK2/STAT5B Pathway

**DOI:** 10.7150/jca.79726

**Published:** 2023-01-01

**Authors:** Qian Liu, Yutao Shen, Yujia Xiong, Jiwei Bai, Yazhuo Zhang, Chuzhong Li

**Affiliations:** 1 Beijing Neurosurgical Institute, Capital Medical University, Beijing 100070, China.; 2 Department of Neurosurgery, Beijing Tiantan Hospital, Capital Medical University, Beijing 100070, China.

**Keywords:** Pituitary adenoma, Estrogen receptor, JAK2, STAT5B, Brain tumor

## Abstract

Total 158 gonadotropin-type pituitary adenoma tissue specimens were collected and the expression of ESR1 in gonadotropin-type pituitary adenoma and its association with the overall survival of patients were analyzed. Transcriptome-sequencing data containing 79 cases of gonadotropin-type pituitary adenoma was used to search for all ESR1-related genes. KEGG pathway enrichment analysis was performed to identify the altering pathway and targeting genes. The *in vitro* and *in vivo* pituitary models were used to evaluate the therapeutic efficacy of estrogen receptor (ER) inhibitors AZD9496 and fulvestrant. The mechanism of AZD9496 and fulvestrant in suppressing pituitary adenoma were also investigated. Low-level ESR1 had longer progression-free survival (PFS) in pituitary adenoma patients. ErbB signaling pathway was discovered as the main enriched pathway. Furthermore, the STAT5B gene was identified as a key ESR-1-related gene. The expression of STAT5B was significantly positively correlated with ESR1 expression in the pituitary adenoma. AZD9496, a novel ER inhibitor, exhibited a potent inhibitory effect on the growth of *in vitro* and *in vivo* pituitary adenoma cells, and its efficacy is comparable to the classic ER inhibitor, fulvestrant. Mechanically, the AZD9496 and fulvestrant significantly blocked JAK2/STAT5B pathway in GT1-1 cells and xenograft mice. Our results provide substantial evidence for the subsequent clinical use of AZD9496 in the treatment of patients with pituitary adenoma.

## Introduction

Due to the high incident of pituitary adenoma, especially prolactin-secreting and incidental lesions, it become the common benign tumors and the 3^rd^ most-common primary intracranial tumors 1. Based on the biological behavior, pituitary adenoma can be classified into benign adenoma, invasive adenoma and carcinoma 2. Although most pituitary adenoma are not cancerous, they can lead the pituitary to produce abnormal hormones, resulting in health problems 3-5. Usually, it causes endocrine metabolic disorders and is harmful to the corresponding target organs. However, this type of tumors can grow and form into large tumors and lead to hypopituitarism and visual disturbances 6.

ERα is known as the dominant isoform in the pituitary adenoma 7. Estrogen, a key regulator of the synthesis and secretion of several pituitary hormones through the activation of ERα. What is more, the expression level of ERα is associated with the poor prognosis in patients with pituitary adenoma 8. Fulvestrant is a pure antiestrogen and can inhibit the growth of GH3 cells (pituitary adenoma). Fulvestrant treatment resulted in an approximately 50% reduction of tumor size 9. Unfortunately, the dose and route of administration (intramuscular injection) of fulvestrant lead to the limitation of its clinical application. Although the recently approved fulvestrant 500 mg monthly dosing regimen has shown data on its effect on ER 10, the efficacy of blockade of ER is still less than 75%. The active metabolites of tamoxifen, 4-OH tamoxifen, and phenolic structure of fulvestrant are similar structures to the estradiol A-ring 11, while the ability of these compounds to bind ER was enhanced, the changes of phenolic structure resulted in a higher clearance and lower bioavailability. Therefore, to obtain compounds without phenol structure, the candidates were re-selected and modified 12, 13. These series of compounds exhibited good ability to bind ER and reduced ER expression.

Among of these designed compounds, AZD9496 with its excellent ERα degradation ability (IC_50_=0.14 nmol·L-1) was discovered 14. There is no significant difference between AZD9496 and fulvestrant in ERα binding and degradation *in vitro*, while the *in vivo* tumor inhibition rate of AZD9496 is higher than that of fulvestrant. Consideration of lower bioavailability of fulvestrant in clinical and animal models, it is speculated that the level of free drug concentration in the xenograft tumor model may lead to the difference in tumor growth inhibition 12, 15. This finding suggests that increasing blood concentration of estrogen inhibitors will contribute to enhancing the degradation of ERα and inhibiting of tumor growth *in vivo*. The good performance of AZD9496 in terms of route of administration and bioavailability has laid the foundation for its follow-up clinical trials.

Phase I clinical trials of AZD9496 were conducted in the United States and the United Kingdom in 2014 and 2015, respectively 16. A phase I clinical trial involving 45 patients showed that AZD9496 has good tolerability and safety. It has the effect of prolonging the stable time of the disease in advanced breast cancer patients with ER, EGFR positive and Her-2 negative. Most recently, researchers found that AZD9496 significantly inhibited the cell proliferation and induced apoptotic cells, suggesting that AZD9496 also has a strong inhibitory effect on pituitary adenoma cells. Therefore, AZD9496 effectively blocked the ERα and suppressed the growth of pituitary adenoma cells GH3 and MMQ 17.

Herein, the association of ESR1 expression and overall survival of patients with pituitary adenoma were analyzed. Transcriptome-sequencing and KEGG pathway enrichment analysis was performed to identify the altering pathway and targeting genes. We will compare the therapeutic efficacy of AZD9496 and fulrestrant in pituitary adenoma models and investigate the potential mechanisms.

## Materials and Methods

### Patients and tissue specimens

In this study, a total of 237 patients with primary gonadotroph pituitary adenoma who received surgical resection at Beijing Tiantan Hospital were included. Patients were included according to the following criteria: (1) the radiological and pathological diagnosis was pituitary adenoma; (2) no evident abnormal showed in the levels of serum hormones; (3) the report of immunohistochemistry only showed positives in FSH and LH; (4) the tumor was primary; (5) complete clinical information. Exclusion criteria: (1) the tumor size was too small to perform RNA sequencing or to build the tissue microarray (TMA); (2) the patient had other malignant tumors; (3) the patient lost the follow-up. Frozen tissue specimens obtained from 79 patients with primary gonadotroph pituitary adenoma who received surgical resection at Beijing Tiantan Hospital between June 2018 and May 2019 were subjected to RNA sequencing. Paraffin-embedded tissue specimens obtained from 158 patients with primary gonadotroph pituitary adenoma who received surgical resection at Beijing Tiantan Hospital between April 2011 and March 2016 were subjected to tissue microarray (TMA). These patients were followed up by radiographical and clinical examinations in July 2020. Tumor recurrence was confirmed by clinical and imaging findings or histology analysis of specimens from the second surgery. This study was approved by the ethics committee of Beijing Tiantan Hospital, Capital Medical University. Informed consent was obtained from all the enrolled subjects, and the study was performed in compliance with the principles governed by the Declaration of Helsinki.

### RNA sequencing and bioinformatics analysis

RNA-seq was performed in 79 gonadotroph pituitary adenoma. A total amount of 3 μg RNA per sample was used as input material for RNA sample preparations. First, ribosomal RNA was removed by Epicentre Ribo-zero rRNA Removal Kit (RZH1046, Epicentre). Subsequently, sequencing libraries were generated using the rRNA-depleted RNA by NEBNext Ultra Directional RNA Library Prep Kit for Illumina (E7420L, NEB) following the manufacturer's protocol. First strand cDNA was synthesized using random hexamer primer. Second-strand cDNA synthesis and making, which incorporates dUTP into the second strand, converts the cDNA. Double-stranded DNA was repaired via exonuclease/polymerase activities and then added adenylation to the 3' end. After adapter ligation and library amplification, the library fragments were purified with AMPure XP system (Beckman Coulter, Beverly, USA) in order to select fragments of preferentially 150-200 bp in length. The strand marked with dUTP is not amplified, allowing strand-specific sequencing. Finally, products were purified (AMPure XP system) and library quality was assessed on the Agilent Bioanalyzer 2100 system. After cluster generation, the libraries were sequenced on an Illumina Hiseq X platform and 150 bp paired-end reads were generated. For RNA-sequenced data analysis, R v3.6.3 software and the LIMMA R package were adopted to screen the genes correlated with ESR1, and the genes significantly correlated with ESR1 (|r| > 0.3, *p* < 0.05) were finally selected.

### TMA construction

Paraffin-embedded gonadotroph pituitary adenoma tissue specimens from all 158 patients were assayed by TMA using the Tissue Array MiniCore (ALPHELYS, Plaisir, France). Three pathologists viewed the hematoxylin-eosin stained slides, and the two most representative 2 mm cores from every tissue slide were selected and removed to a new slide to build the TMA. The 4 μm sections from the TMA were cut using Leica RM 2135 Rotary Microtome (Rankin, Wetzlar, Germany) for immunohistochemical staining.

### Immunohistochemistry (IHC)

The slides were placed in the BOND-III instrument manufactured by Leica Biosystems. Default IHC protocol was chosen, and 20 min with epitope retrieval was set as the heat-induced epitope retrieval (HIER) parameter. The Bond™ Polymer Refine Detection (DS9800, Leica Biosystems, Germany) was used for the detection of the primary antibody (anti-ESR1 antibody, MA1-80216, Thermo Fisher, USA). The slides were scanned using Aperio AT2 (Leica Biosystems, Germany) and the digital pictures were viewed using digital slide viewing software in Aperio AT2. The staining intensity was stratified on a scale of 0-3+ (0 = no staining, 1+ = weak, 2+ = moderate and 3+ = strong). An H-score was obtained by multiplying the staining intensity with a constant to adjust the mean to the strongest staining [H-score = 1 × (percent of 1+ cell) + 2 × (percent of 2+ cell) + 3 × (percent of 3+ cell)] to give a score ranging from 0-300. We chose the median of the H-scores as the cut-off value for separating patients into two groups: high ESR1 expression or low ESR1 expression.

### Cell culture and pharmacological treatment

The rat gonadotroph adenoma cell line GT1-1 and rat PA cell line GH3 were purchased from the American Type Culture Collection (ATCC) and cultured in DMEM Medium (11995-065, Gibco, USA) supplemented with 10% fetal bovine serum (FBS, 10099-141, Gibco, USA) in a humidified incubator at 37 °C in 5% CO_2_. The culture medium was replaced every other day. The human primary gonadotroph adenoma cells were isolated from gonadotroph adenoma tissues. The fresh gonadotroph adenoma tissues were obtained at the time of surgery and transferred in L15 medium enriched with 10% FBS. The tumor tissues were cut into small pieces and then were digested with collagenase (1 mg/mL, 17101015, Thermo Fisher, USA) at 37 °C for 30 minutes. After terminating the enzymatic treatment by addition of FBS, the mixture was filtered with cell strainer to remove undigested tissues and centrifuged at 600 rpm for 5 minutes. The cell pellet was resuspended in Neurobasal growth medium supplemented with 2% B27 (A3582801, Thermo Fisher, USA) and plated on 35 mm dishes. Tumor cells were infected with adenovirus at MOI of 30 or 100 for 48 hours. Tumor cells were digested and centrifuged for 5 minutes and suspended in the medium. Live cells were calculated and re-plated in 24-well plates. For estrogen receptor degradation, the GH3 cells, GT1-1 cells and primary gonadotroph adenoma cells were treated with 25nM, 50nM, 100nM, and 200nM AZD9496 (HY-12870, MCE, USA) or Fulvestrant(HY-13636, MCE, USA) for 1-4 days.

### Cell viability and proliferation assay

Cell viability was determined using a Cell Counting Kit-8 (CK04, Dojindo, Japan). The GH3 cells, GT1-1 cells and primary gonadotroph adenoma cells were seeded into 96-well plates at approximately 1 × 10^4^ cells per well. Different concentration of AZD9496 and Fulvestrant was then added into the wells and incubated for 24, 48, 72 and 96 hours. Upon addition of CCK-8 solution, the plates were incubated at 37°C for 3 hours, and the absorbance was detected at 450 nm using a multimode microplate reader (Tecan, Männedorf, Switzerland). Cell proliferation was determined using a BrdU Cell Proliferation ELISA (11647229001, Roche, Switzerland) according to the manufacturer's instructions. The GT1-1 cells were seeded into 96-well plates at approximately 1 × 10^4^ cells per well. Different concentration of AZD9496 and Fulvestrant was then added into the wells and incubated for 24, 48, 72 and 96 hours. Upon addition of BrdU solution, the plates were incubated at 37°C for 2 hours, then the cells were fixed for 30 minutes at room temperature. Subsequently, upon addition of anti-BrdU-POD solution, the plates were incubated at room temperature for 90 minutes. After Substrate solution and 1M H_2_SO_4_ were added into the wells, the absorbance was detected at 450 nm using a multimode microplate reader (Tecan, Männedorf, Switzerland).

### RNA extraction and qRT-PCR

Total RNA was extracted using RNeasy Mini Kit (74104, Qiagen, Germany), and reverse transcription was performed using High Capacity cDNA Reverse Transcription Kit (4368814, Thermo Fisher, USA) according to the manufacturer's protocol. Subsequently, we performed qRT-PCR using Power SYBR Green PCR Master Mix (4367659, Thermo Fisher, USA) in a total reaction volume of 20 μL. GAPDH was used as a reference gene. The levels of mRNAs were performed on a QuantStudio 5 (Applied Biosystems, USA). Amplification was performed as follows: 95 °C for 10 minutes and 40 cycles at 95 °C for 15 seconds, 60 °C for 60 seconds. For the quantitative analysis, relative expression levels were calculated based on CT values (corrected for GAPDH expression) according to the equation: 2^-ΔCT^[ΔCT = CT (gene of interest) - CT (GAPDH)]. All qRT-PCR analyses were performed in triplicate. The primer sequences are provided in Table S3.

### Protein extraction and Western Blot assay

Tissue samples or cells were lysed using RIPA lysis buffer (C1050, Applygen, China) with a protease inhibitor cocktail (P1265, Applygen, China) and a phosphatase inhibitor cocktail (P1260, Applygen, China). The total protein concentration was determined using a BCA Protein Assay Kit (SK258437, Thermo Fisher, USA). Equal amounts of total proteins were separated by SDS-PAGE (10% gels) for JAK2, phospho-JAK2, STAT5B, and phospho-STAT5B detection. GAPDH was used as the protein loading control. After SDS-PAGE, the proteins on the gels were transferred to BioTrace nitrocellulose membranes (66485, Pall, USA), blocked with 5% skim milk in Tris-buffered saline (TBS, pH 7.4; 20 mM Tris-HCl, 150 mM NaCl), and then incubated with anti-JAK2 antibody (1:1000, 3230, CST, USA), anti-phospho-JAK2 antibody (1:1000, 3776, CST, USA), anti-STAT5B antibody (1:800, MA5-15665, Thermo Fisher, USA), and anti-phospho-STAT5B antibody (1:600, 33-6000, Thermo Fisher, USA) overnight at 4 °C. The following day, the membranes were incubated with IRDye-labeled goat anti-rabbit or goat anti-mouse IgG at room temperature for 1 hour. Finally, the protein bands were scanned using a Li-COR Odyssey system (Li-COR Biosciences, USA). At least three independent experiments were performed, and a representative result is shown.

### Xenograft mice model

To establish the gonadotroph adenoma model, 2 × 10^6^ GT1-1 cells were suspended in 100 μL PBS and then subcutaneously injected into the left axilla of BALB/c nude mice (4 weeks of age, female; Beijing Vital River Laboratory Animal Technology, China). The mice started to receive AZD9496 or Fulvestrant treatment when the diameter of the tumors reached 3 mm. AZD9496 was given to the mice by gavage, and Fulvestrant was given to the mice by intramuscular injection. For AZD9496 treatment, the mice were dosed daily with the concentration of 0.1mg/kg, 0.5mg/kg and 5mg/kg. For Fulvestrant treatment, the mice were dosed twice per week with the concentration of 0.5mg, 3mg and 20mg per mouse. After 2 weeks of treatment, the tumor xenografts were extracted, weighed and applied to RNA extraction and protein extraction. Tumor volume was determined using the following formula: volume = (length × width^2^)/2.

### Statistical analysis

Data are presented as the mean ± SEM. Statistical analyses were performed using SPSS v24.0 software (IBM Corporation, USA). Variables were analyzed by the chi-square test, Fisher's exact test, unpaired Student's *t* test, or Mann-Whitney *U* test for comparison between two groups. Kaplan-Meier curves and the log-rank test were applied for univariable survival analysis. For all statistical analyses, a *p* value less than 0.05 was considered statistically significant.

## Results

### Expression of ESR1 in gonadotropin-type pituitary adenoma and its association with progression-free survival of patients

**Figure 1:**
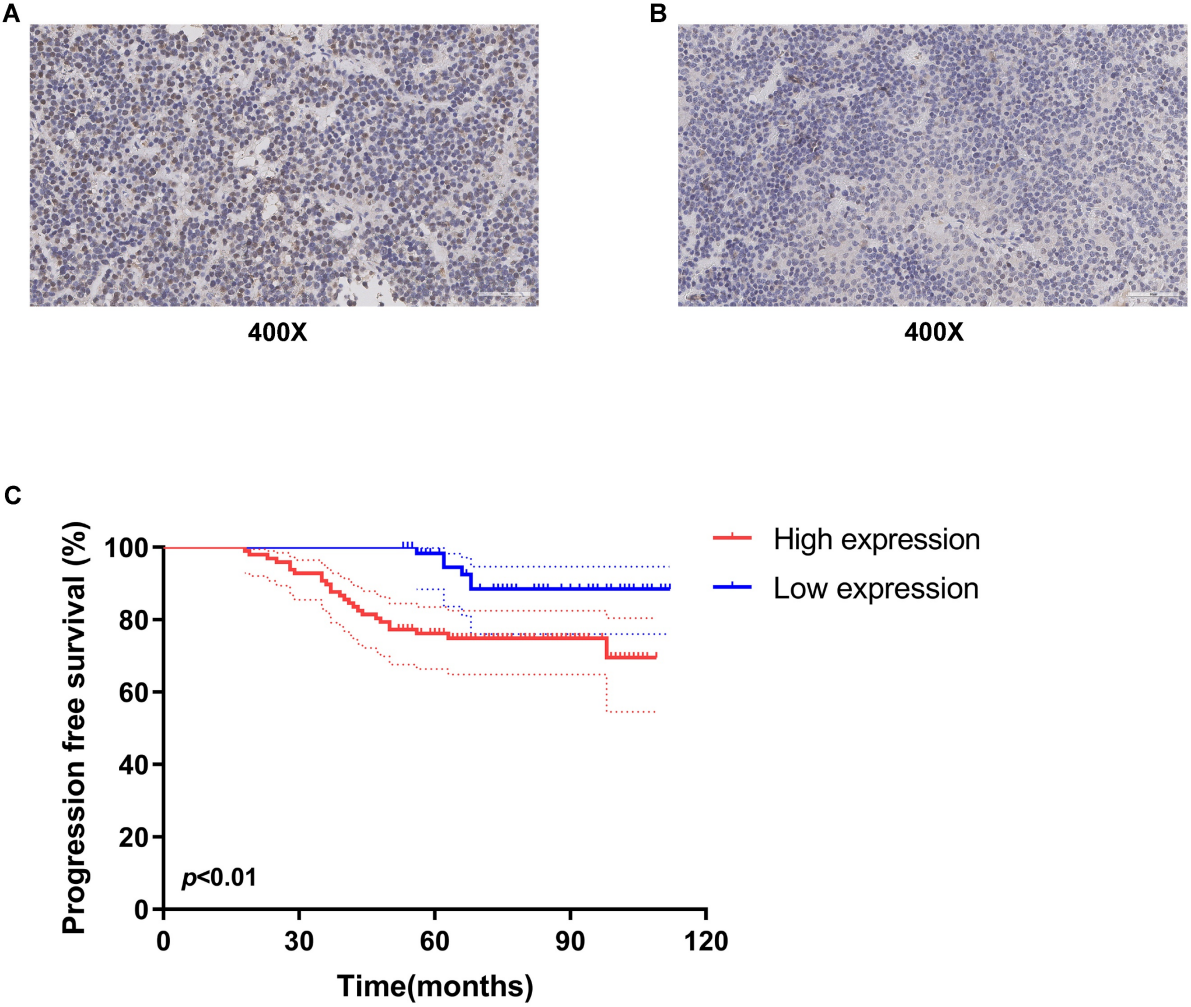
** Expression of ESR1 in gonadotropin-type pituitary adenoma and its association with overall survival of patients. Representative images of ESR1 immunohistochemical staining in gonadotroph adenomas. (A)** High expression of ESR1 (Left, magnification: x400) and **(B)** low expression of ESR1 (Right, magnification: x400); **(C)** analysis of progression free survival (PFS) using Kaplan-Meier survival curves. low-level expression of ESR1 was correlated with longer PFS, *p* < 0.01.

To evaluate the ESR1 expression, the 158 gonadotropin-type pituitary adenoma tissue specimens were collected from patients who underwent tumor resection at Beijing Tiantan Hospital from April 2011 to March 2016. The clinical characteristics of these 158 patients were listed in Table 1. The follow-up information of these patients was updated to July 2020. Furthermore, the expression level of ESR1 in gonadotropin-type pituitary adenoma was measured and quantified by using immunohistochemical staining. As shown in Figure 1A&B, ESR1 protein was expressed in tumor cell nuclei, and the H-scores of its expression ranged from 93.57 to 210.83 (median 145.14). Based on the median of the H-scores, cutoff value was selected, then 158 patients were divided into two groups: high-level and low-level ESR1 expression. There were 97 patients with high-level expression and 61 patients with the low expression of ESR1. We first analyzed the association between ESR1 expression and clinical characteristics. There was no significant difference in the distribution of males and females in the two groups (*p* = 0.66). The mean age of patients was 52.3 years (range 21-75 years) in the high-level ESR1 group and 47.1 years (range 15-71 years) in the low-level ESR1 group (*p* = 0.25). The mean tumor volume in the high expression group was 35.2 cm^3^ (range 2.1-256.4 cm^3^), while in the low expression group, the mean tumor volume was 29.6 cm^3^ (range 2.8-202.5 cm^3^) (*p* = 0.19). For the levels of serum hormones, no significant difference was observed between the two groups (*p* = 0.45, 0.82, 0.60, 0.53 and 0.51, respectively). Moreover, there was no remarkable difference in the recurrence rate between the two groups (*p* = 0.74). 66/97 of patients received TR/STR in the high-level ESR1 group and 48/61 of patients in the low-level ESR1 group (*p* = 0.16). And the tumor was invasive in 72 patients and non-invasive in 86 patients, no significant difference was observed between the two groups (*p* = 0.38). Progression-free survival (PFS), as the time from a patient's first tumor resection until tumor recurrence or last follow-up, was used to survival analysis. We found that the median PFS of all patients is 75.5 months. The median PFS of patients in the ESR1 low-expression and high-expression group was 84 months and 69 months, receptively. Median FPS in Low ESR1group is 1.2-fold higher than that in ESR1 high-level group. Noticeably, patients with low ESR1 expression had longer PFS (median 84 months, 95% confidence interval 79.4-89.1 months) (Figure 1C).

### Effect of AZD9496 and fluvestrant on the growth of GT1-1, GH3 and primary gonadotroph adenoma cells

**Figure 2:**
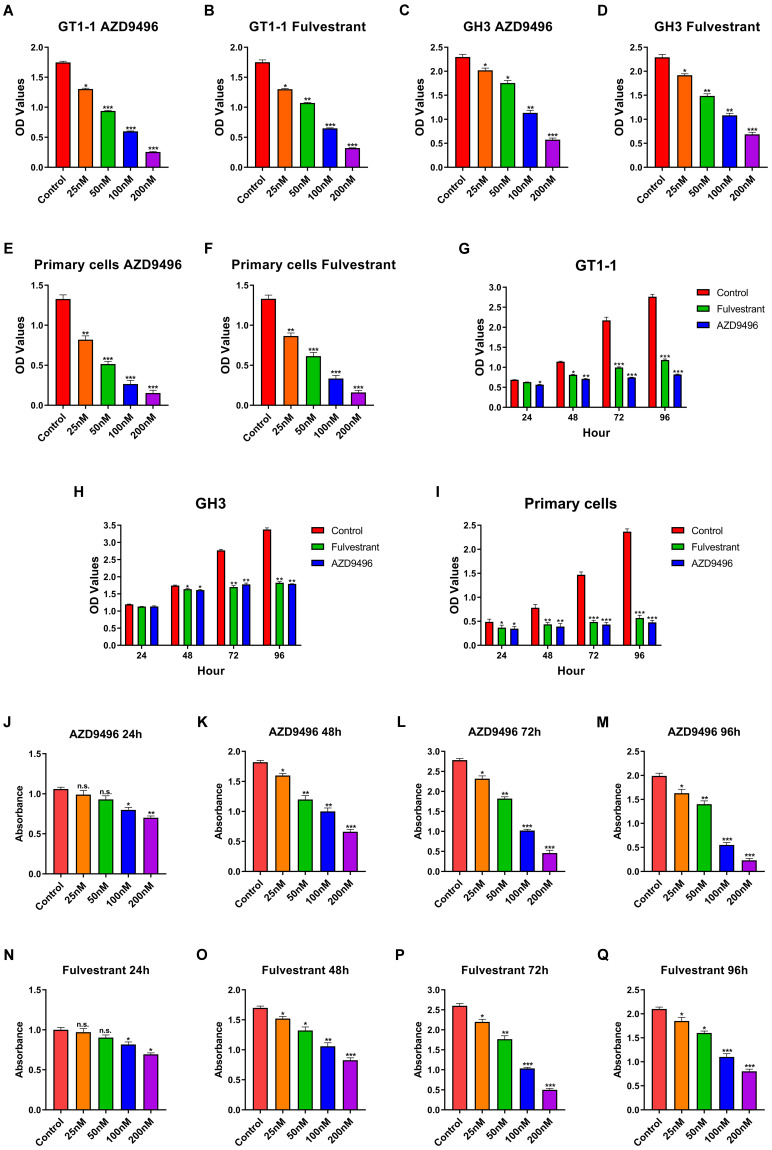
** Effect of AZD9496 and fluvestrant on the growth of GT1-1, GH3 and primary gonadotroph adenoma cells. (A&B)** The cell viability of GT1-1 cells was inhibited after 72 hours AZD9496 and fulvestrant treatment; **(C&D)** The effect of AZD9496 and fulvestrant on the growth of GH3 cells;** (E&F)** The effect of AZD9496 and fulvestrant on the growth of primary gonadotroph adenoma cells; **(G-I)** The effect of AZD9496 (100nM) and fulvestrant (100nM) on the growth of GT1-1, GH3 and primary cells at 24, 48, 72 and 96 hours; **(J-N)** The dose- and time-dependent manner of AZD9496 and fulvestrant on suppressing the growth of GT1-1 cells was measured by BrdU assay. Bars represent the mean of the respective individual ratios ± SEM. *p < 0.05; **p < 0.01; ***p < 0.001; n.s, not significant.

We next compare the effect of the novel ER inhibitor AZD9496 and fulvestrant (the classic ER inhibitor) on the growth of pituitary adenoma cells and primary gonadotroph adenoma cells. GH3, GT1-1 and primary cells were treated by different concentrations of AZD9496 or fulvestrant (0, 25, 50, 100, 200nM) for 72 hours. Cell growth was determined by CCK-8 assay. As shown in Figure 2A&B and 2C&D, the cell viability of GT1-1 and GH3 cells were significantly suppressed by ER inhibitors in a dose-dependent manner. The IC_50_ of AZD9496 and fulvestrant is about 50 nM in GT1-1 cells, and 100 nM in GH3 cells. GT1-1 cells exhibited more sensitive to ER inhibitors than GH3 cells. In addition to pituitary adenoma cells, the effect of AZD9496 and fulvestrant on the growth of primary gonadotroph adenoma cells were also determined. As the results shown in Figure 2E&F, after 72 hours treatment, the two ER inhibitors exhibited the most potent effect on inhibiting the proliferation of gonadotropin-type pituitary adenoma primary cells.

In addition to the dose-dependent manner, we also investigated the effect of AZD9496 and fulvestrant on the cell growth in a time-dependent manner. Pituitary adenoma cells GT1-1, GH3 and primary gonadotroph adenoma cells were treated by AZD9496 (100nM) and fulvestrant (100nM) for 24, 48 and 72 hours, the cell proliferation was determined by CCK-8 assay. As seen in Figure 2G-I, after 48 hours treatment of ER inhibitors, the proliferation of GT1-1 and GH3 were significantly suppressed. Interestingly, the significantly inhibition of primary gonadotroph adenoma cells was observed at 24 hours.

In addition to CCK-8 assay, the effect of AZD9496 and fulvestrant on the growth of GT1-1, GH-3 and primary gonadotroph adenoma cells were determined by BrdU cell proliferation assay. As the results shown in Figure 2J-Q, the ER inhibitors (both AZD9496 and fulvestrant) could effectively inhibit the proliferation of these cells.

### Effect of AZD9496 and fulvestrant on suppressing JAK2/STAT5B pathway in GT1-1 cells

**Figure 3:**
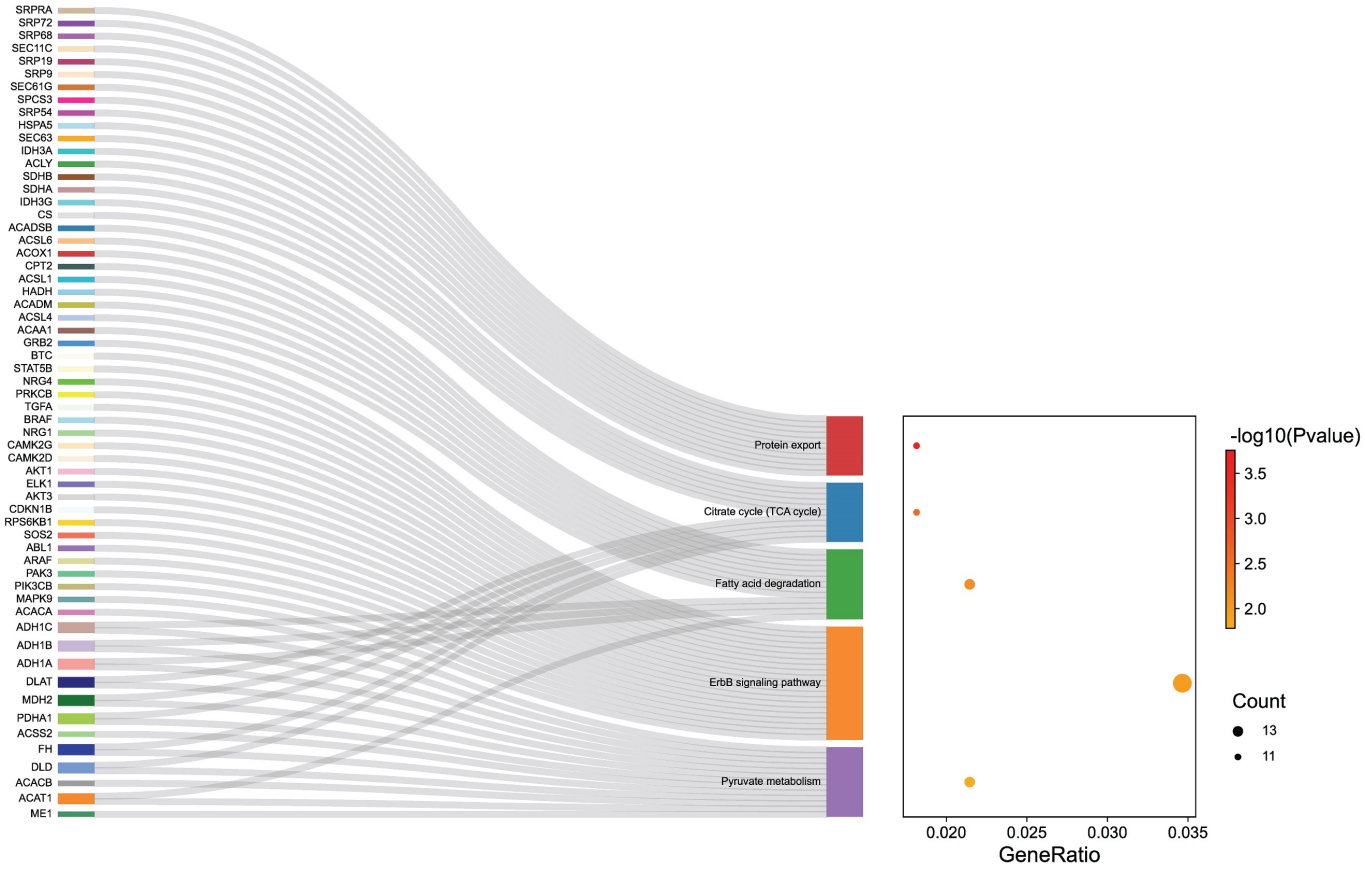
Transcriptome data of gonadotroph adenomas revealed that the ESR1-related genes were enriched in the ErbB signaling pathway.

**Figure 4:**
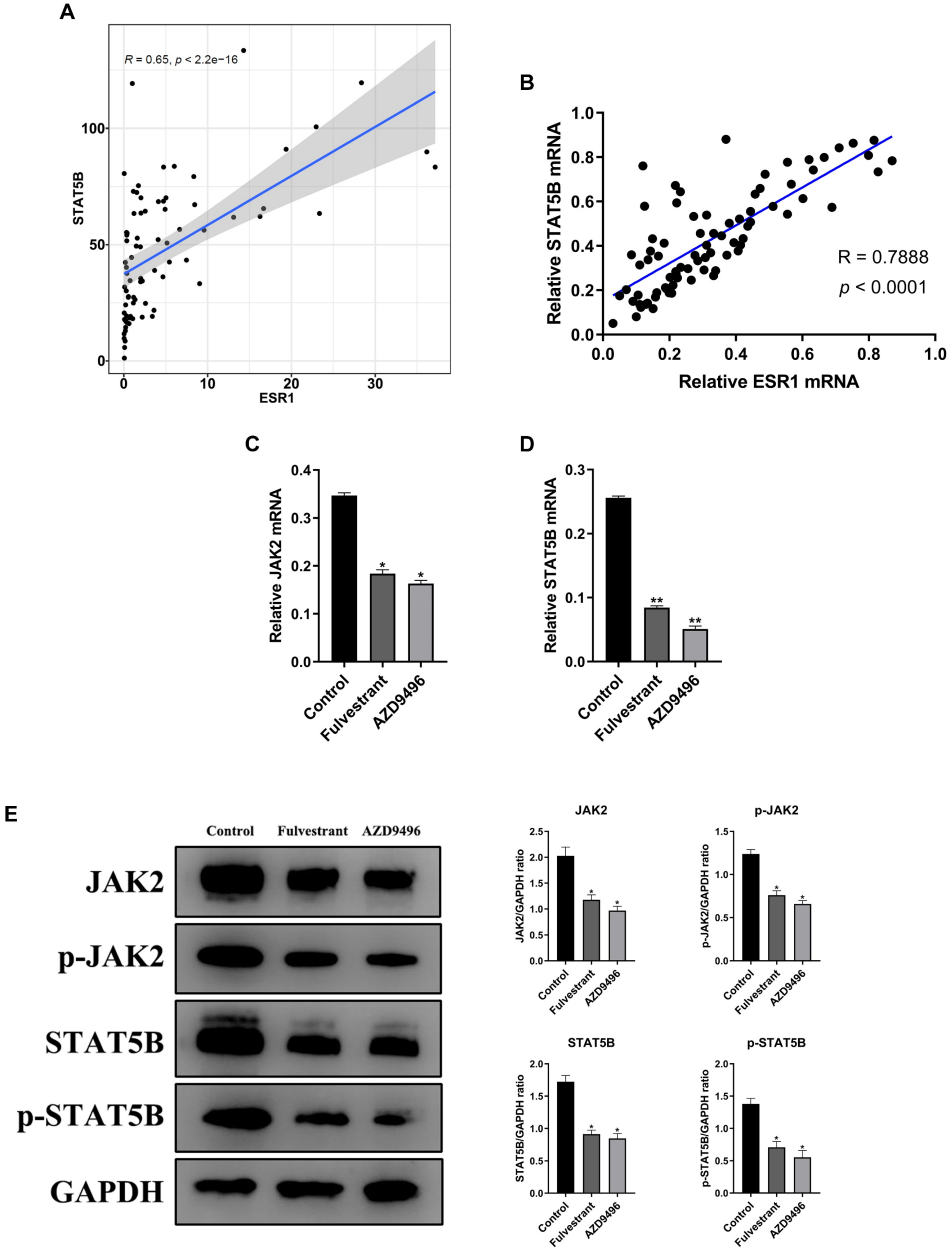
** Effect of AZD9496 and fulvestrant on blocking JAK2-STAT5B pathway in pituitary adenoma cells. (A)** Transcriptome data of gonadotroph adenomas showed that there was a positive correlation between the expression of ESR1 and the expression of STAT5B; **(B)** The mRNA expression of ESR1 and STAT5B showed a significantly positive correlation; **(C&D)** Effect of AZD9496 and fulvestrant on the mRNA level of JAK2 and STAT5B. The alteration of JAK2 and STAT5B mRNA was determined by real-time PCR; **(E)** Western Blot assay verified that the inhibition of ESR1 resulted in the reduction of JAK2 and STAT5B in GT1-1 cells. Bars represent the mean of the respective individual ratios ± SEM. *p < 0.05; **p < 0.01.

To explore the potential mechanisms of ER inhibitors on suppressing the growth of gonadotropin-type pituitary adenoma, a transcriptome-sequencing analysis was performed. We first analyzed transcriptome-sequenced data containing 79 cases of gonadotropin-type pituitary adenoma, searching for all ESR1-related genes which are associated with gonadotropin-type pituitary adenoma (Table S1). KEGG pathway enrichment analysis was performed to identify the altering pathway and targeting genes. ErbB signaling pathway was discovered as the main enriched pathway (Figure 3, Table S2). Furthermore, in ErbB signaling pathway, the STAT5B gene was identified as a key ESR1-related gene in the gonadotropin-type pituitary adenoma (Table S2). What is more, further studies suggested that the expression of STAT5B was significantly positively correlated with the expression level of ESR1 (Figure 4A, Table S1). To confirm these finding, total RNA was extracted from 79 gonadotropin-type pituitary adenoma samples, the mRNA expression of ESR1 and STAT5B was determined by RT-PCR. Correlation analysis demonstrated that the mRNA expression level of STAT5B and ESR1 did show a significantly positive correlation (Figure 4B). Our results are consistent to the bioinformatics predication.

Given that JAK2 gene is an important upstream regulator of STAT5B gene 18, we speculate that ER inhibitors affect the JAK2-STAT5B pathway in pituitary adenoma cells. The total RNA and total protein lysis were extracted from ER inhibitors-treated GT1-1 cells, and the expression levels of JAK2 and STAT5B at the mRNA and protein levels were determined by RT-PCR and immunoblot analysis, respectively. As seen in Figure 4C&D, the mRNA level of JAK2 and STAT5B were markedly suppressed by ER inhibitors. Consistently, after treatment with AZD9496 or fulvestrant, the expression of total JAK2 and STAT5B were significantly decreased, and the expression of p-JAK2 protein and p-STAT5B protein were also reduced. (Figure 4E). These results confirmed that AZD9496 and fulvestrant effectively blocked theJAK2-STAT5B pathway in GT1-1 cells.

### Effect of AZD9496 and fulvestrant on pituitary adenoma growth *in vivo*

To further validate the effect of AZD9496 and fulvestrant on the growth of the pituitary adenoma, GT1-1 cells xenograft mice model was established. 2x10^6^ GT1-1 cells were subcutaneously implanted into BALB/c nude mice and administration of ER inhibitors was performed once the tumor size reach 3 mm in length. As seen in Figure 4A&B, administration of AZD9496 (0.1, 0.5, and 5mg/kg) significantly suppressed the pituitary adenoma growth in a dose-dependent manner. 0.1mg/kg of AZD9496 alone inhibited over 60% of tumor volume, as compared the control group. Meanwhile, the administration of fulvestrant (0.5, 3 and 20 mg/mouse) was used as a positive control. As the results shown in Figure 4A&C, fulvestrant also significantly inhibited the growth of pituitary adenoma. In addition to the tumor volume, the tumor mass was also weighed and quantified. Consistent with the tumor volume, the ER inhibitors effectively suppressed the tumor weight *in vivo* (Figure 4D&E). To confirm the mechanism of ER inhibitor in suppressing pituitary adenoma growth *in vivo*, the mRNA and protein expression of JAK2 and STAT5B in GT1-1 xenograft tumor tissues were determined by qRT-PCR and immunoblot analysis, respectively. After treatment with different doses of AZD9496 or fulvestrant, the expression of JAK2 and STAT5B mRNAs were obviously decreased in a dose-dependent manner (Figure 4F&G), and the expression of p-JAK2, p-STAT5B and total JAK2 and STAT5B proteins were also significantly reduced in a dose-dependent manner (Figure 4H). Similarly, biological response to AZD9496 and fulvestrant was observed in GT1-1 cells xenograft mice model. 20 mg/mouse of fulvestrant or 5 mg/kg of AZD9496 completely blocked p-JAK2, JAK2, and strongly inhibited p-STAT5B and STAT5B expression. Moreover, this result was consistent with previous observation in pituitary adenoma cellular model. Taken together, our *in vivo* mice model confirmed that AZD9496 and fulvestrant can inhibit the growth of gonadotropin-type pituitary adenoma by inhibiting the JAK2-STAT5B pathway.

## Discussion

Usually, ER is mainly expressed in the lung, bone, kidney, uterus, mammary glands and urogenital tract cells 19-23, and administrates the estrogen-mediated proliferation, differentiation and development of reproductive tissues 24-26. ERα is known as the dominant isoform in the pituitary adenoma 27. In this study, the expression level of ESR1 and the relationship of ESR1 and overall survival of patients with pituitary adenoma were investigated. Our results showed that low level of ESR1 expression had a 1.2-fold longer PFS. This finding suggests that ESR1 expression may be play an important role in the cell proliferation and differentiation of pituitary adenoma cells.

Fulvestrant, a classic estrogen receptor antagonist, was used as a hormone treatment of HR-positive metastatic breast cancer in post-menopausal women with disease progression following anti-estrogen therapy 28. AZD9496, a nonsteroidal small-molecule inhibitor of ERα, is a potent and selective antagonist and degradation of ERα *in vitro* and *in vivo* in ER-positive models of breast cancer 29, 30. The effect of fulvestrant and AZD9496 on the proliferation of ESR1-postive pituitary adenoma were investigated. Fulvestrant and AZD9496 exhibited potent anti-proliferative activity against GT1-1 and GH-3 cells. What is more, the more sensitive response to ER inhibitors were observed in primary pituitary adenoma cells. This result suggests that blockage of ER could effectively suppress the growth of pituitary adenoma growth. In addition to *in vitro* model, the ability of AZD9496 and fulvestrant bind ER and degrade ER *in vivo* was compared. In terms of degradation, AZD9496 is comparable to fulvestrant in potency. The anti-tumor efficacy of AZD9496 (administered 5mg/kg, q.d.) *in vivo* is better than that of fulvestrant (administered 20mg/mouse, twice/week), and the inhibition rate of tumor growth reaches 75%.

To clarify the potential mechanism of ER-driven growth of pituitary adenoma, Transcriptome-sequencing and KEGG pathway enrichment analysis was performed to identify the altering pathway and targeting genes. JAK2/STAT5B pathway was identified as a key biological event in ER-mediated growth of pituitary adenoma. STAT5B belongs to the seven STAT family members, and it can be activated by multiple biological events, including cytokines, hormones, and growth factors. For example: Interleukin-6, epidermal growth factor (EGF), and growth hormones 31-33. JAK/STAT pathway takes part in the pathogenesis of multiple solid cancers and hematopoietic malignancies 34-36. Especially, constitutive activation of STAT5B is involved in progression in multiple cancers, it has been shown to be involved in the survival, proliferation, and differentiation of immune cells and hematopoietic cells 37-39. In this study, we found the STAT5B is positively related to ESR1 expression in pituitary adenoma. It suggests that STAT5B may be a key player in the ESR1-reuglated growth of pituitary adenoma. Our further study confirmed that fulvestrant and AZD9496 significantly blocked the mRNA and protein level of STAT5B *in vitro* and *in vivo* pituitary adenoma model. JAK2 as the upstream regulator were also investigated. Consistently, the ER inhibitors also effectively suppressed JAK2 expression.

In summary, our immunohistochemical staining result from the specimen samples demonstrated that low-level ESR1 had longer progression-free survival (PFS) in pituitary adenoma patients. Transcriptome-sequencing and KEGG pathway enrichment analysis suggests STAT5B gene can be served as a key ESR1-related gene. Further studies showed that STAT5B was significantly positively correlated with ESR1 expression in pituitary adenoma. AZD9496, a novel ER inhibitor, exhibited a potent inhibitory effect on the growth of *in vitro* and *in vivo* pituitary adenoma cells, and its efficacy is comparable to the classic ER inhibitor, fulvestrant. Both AZD9496 and fulvestrant could significantly block JAK2/STAT5B pathway in pituitary adenoma models. Our findings provide substantial evidence for the potential clinic usage of AZD9496 in the treatment of patients with pituitary adenoma.

## Supplementary Material

Supplementary tables.

## Figures and Tables

**Figure 5:**
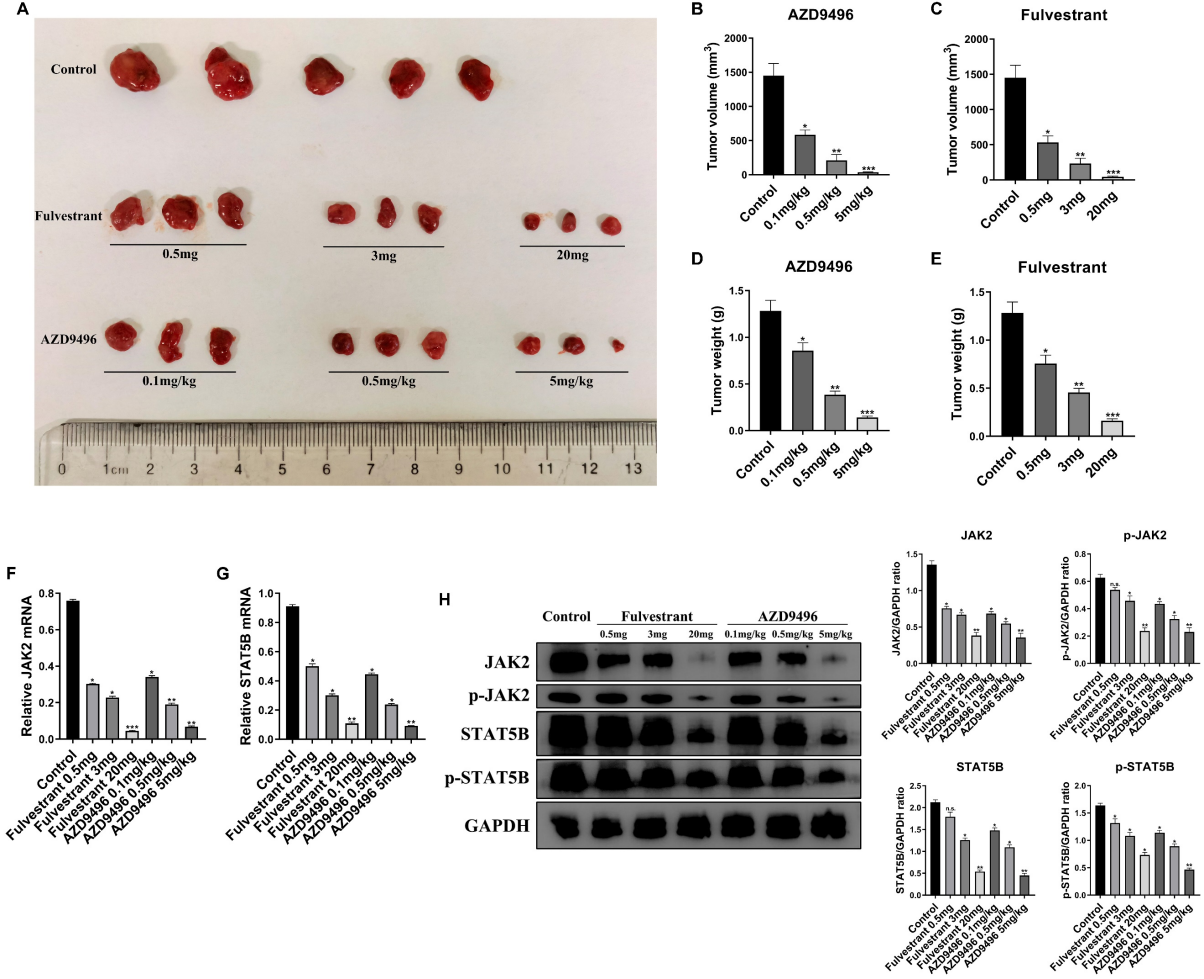
** Effect of AZD9496 and fulvestrant on pituitary adenoma growth *in vivo*. (A)** After 2 weeks of treatment, a significant shrinkage of the xenograft tumors was observed in the fulvestrant group and AZD9496 group compared with the untreated group; **(B&C)** Effect of AZD9496 and fulvestrant on the tumor volume of GT1-1 xenograft mice. Treatment of AZD9496 and fulvestrant treatment for 2 weeks, the tumor volume was a significantly decreased, as compared to the control group; **(D&E)** Effect of AZD9496 and fulvestrant on the tumor weight of GT1-1 xenograft mice. Treatment of AZD9496 and fulvestrant treatment for 2 weeks, the tumor weight was significantly reduced, as compared to the control group; **(F&G)** Effect of AZD9496 and fulvestrant on the mRNA level of JAK2/STAT5B pathway *in vivo*. The alteration of JAK2 and STAT5B mRNA was determined by real-time PCR; **(H)** Effect of AZD9496 and fulvestrant on JAK2/STAT5B pathway *in vivo*. ER inhibitors reduced expression of p-JAK2, p-STAT5B and total JAK2 and STAT5B in the xenograft tumor models. Bars represent the mean of the respective individual ratios ± SEM. *p < 0.05; **p < 0.01; ***p < 0.001; n.s, not significant.

**Table 1:** Patient characteristics

Characteristic	Total	ESR1 expression		P value
		High	Low	
**Sex**				0.66
Male	83	51	32	
Female	75	46	29	
Age, mean (range)	50.3 (15-75)	52.3 (21-75)	47.1 (15-71)	0.25
Tumor volume (cm^3^), mean (range)	32.7 (2.1-256.4)	35.2 (2.1-256.4)	29.6 (2.8-202.5)	0.19
**Hormone**				
PRL (ng/ml)	17.83 ± 7.64	19.48 ± 9.63	16.29 ± 5.54	0.45
HGH (ng/ml)	1.08 ± 0.72	1.15 ± 0.91	1.03 ± 0.64	0.82
TSH (μIu/ml)	1.86 ± 1.14	1.69 ± 0.86	1.95 ± 1.37	0.60
FSH (mIu/ml)	14.21 ± 10.46	14.87 ± 11.17	13.38 ± 9.74	0.53
LH (mIu/ml)	4.93 ± 4.22	4.14 ± 3.73	5.62 ± 4.81	0.51
**Recurrence**				0.74
Yes	17	11	6	
No	141	86	55	
**Extent of resection**				0.16
Total resection/Subtotal resection	114	66	48	
Partial resection	44	31	13	
**Invasion**				0.38
Yes (Knosp ≥ III)	72	46	26	
No (Knosp ≤ II)	86	51	35	
