# Supplementary material for: Comparative Efficacy of AZD9496 and Fulvestrant on the Growth of Pituitary Adenoma via Blocking JAK2/STAT5B Pathway
Source: J Cancer. 2023 Jan 1;14(1):61–71. doi: 10.7150/jca.79726 (PMC9809331; doi:10.7150/jca.79726)
Supplement: Supplementary file 1 — Supplementary tables. [file jcav14p0061s1.pdf]

Supplementary Table 1. Genes significantly correlated with ESR1 (|r| > 0.6, $p < 0.05$ listed)

Gene ID	Correlation coefficient	P value
ASB15	0.759354636	5.04E-16
VASH2	0.741019512	5.82E-16
AC135731.1	0.737238254	9.40E-16
DTNBP1	0.735879102	1.11E-16
AC040963.1	0.717096943	1.06E-16
PCYT1B	0.715239428	1.31E-16
SEL1L2	0.705346301	3.95E-16
AL359962.1	0.699443516	7.48E-16
AC093698.2	0.694018459	1.33E-16
AC124657.1	0.692574492	1.54E-16
ANKRD31	0.690592629	1.89E-16
C2orf50	0.676159896	8.03E-16
LINC01250	0.67264971	1.13E-16
SAP30L	0.67133282	1.28E-16
WDR63	0.668790499	1.63E-16
RAP2C-AS1	0.668259977	1.71E-16
GCNT4	0.661077687	3.34E-16
TMEM163	0.65599848	5.31E-16
STAT5B	0.654688757	2.20E-16
AC006453.5	0.650848128	8.40E-16
TMCC2	0.645903406	1.30E-15
AC233976.1	0.64487106	1.42E-15
GAS1RR	0.644835726	1.42E-15
CRYGB	0.643045547	1.66E-15
AL445623.2	0.642713814	1.71E-15
MID2	0.642349617	1.76E-15
LINC00635	0.63787835	2.57E-15
TTC33	0.635650039	3.10E-15
PDYN-AS1	0.635527335	3.14E-15
PELO	0.635362082	3.18E-15
RPL12P17	0.634932037	3.30E-15
AL365295.1	0.634436907	3.43E-15
CDH12	0.633778168	3.63E-15
KCNH3	0.626240356	6.73E-15
C5orf24	0.625679846	7.04E-15
HIST2H2BB	0.623213987	8.58E-15
LRRC9	0.621175991	1.01E-14
FAM172A	0.620903299	1.03E-14
FAM111A-DT	0.61706517	1.40E-14
TMSB15B	0.616415533	1.47E-14
TBC1D25	0.61493353	1.65E-14
ELL2	0.613066657	1.90E-14
BRD2	0.612192943	2.04E-14
EFHC2	0.611720127	2.11E-14
AC074133.1	0.610484598	2.32E-14
ZNF831	0.609135449	2.57E-14
L3MBTL3	0.609095795	2.58E-14

BEND6	0.60825488	2.75E-14
CXorf40A	0.607052467	3.01E-14
AC104123.1	0.605576719	3.37E-14
SLC27A6	0.605499001	3.39E-14
CALCOCO2	0.605308576	3.44E-14
TIMD4	0.605080967	3.50E-14
KIF2A	0.605079999	3.50E-13
ZNF37A	0.603936686	3.81E-13
FGF12-AS2	0.603607787	3.90E-13
PPIAP80	0.60255969	4.22E-13
FEM1C	0.602379219	4.28E-13
DIMT1	0.601875981	4.44E-13
HEATR4	0.60177573	4.47E-13
GRM1	0.6013547	4.62E-13
EMBP1	0.600509126	4.91E-13
THEGL	0.600447102	4.94E-11

Supplementary Table 2. KEGG pathway enrichment analysis of ESR1-related genes ($p < 0.05$ listed)

ID	Description	P value	Gene ID
hsa03060	Protein export	0.000175868	SEC63/HSPA5/SRP54/SPCS3/SEC61G/SRP9/ SRP19/SEC11C/SRP68/SRP72/SRPRA
hsa03018	RNA degradation	0.000304445	MTREX/DIS3/XRN2/DDX6/TENT4A/HSPA9/ CNOT6/TENT4B/CNOT1/LSM8/CNOT6L/PARN/ HSPD1/PABPC3/PFKM/CNOT8/DIS3L/PABPC5/DHX36/ PABPC1L2B/PABPC1L2A/TTC37/CNOT7/PABPC4L
hsa03015	mRNA surveillance pathway	0.000625019	PPP2R5A/SMG6/PPP2R3A/PPP2R2C/PAPOLA/PPP2R3C/ CSTF2/PPP2CB/CASC3/RNGTT/PPP2R5D/PAPOLG/ GLE1/ETF1/UPF3B/PPP2R1B/PABPC3/PELO/MSI2/ PPP2R5E/NUDT21/PABPC5/PABPC1L2B/ PABPC1L2A/PPP1CC/GSPT2/PABPC4L
hsa04068	FOXO signaling pathway	0.001142769	MAPK9/PIK3CB/ARAF/NLK/SIRT1/SOS2/RBL2/SGK3/PRKAG2/ TGFBR1/CDKN1B/MAPK14/SOD2/AKT3/PRKAB2/PRKAA1/MDM2/ GABARAPL1/PDPK1/AKT1/SKP2/PLK2/ATG12/FBXO25/FOXO1/ HOMER1/GRM1/BRAF/PRKAA2/IRS1/RAG2/GRB2/FOXO4
hsa03022	Basal transcription factors	0.001447362	TAF2/GTF2H1/TBP/CDK7/CCNH/TAF5L/TAF8/GTF2B/ GTF2A2/TAF1/GTF2E1/GTF2A1/TAF7/GTF2H2C/TAF9B
hsa00020	Citrate cycle (TCA cycle)	0.002636127	CS/IDH3G/SDHA/DLD/FH/SDHB/ACLY/PDHA1/MDH2/DLAT/IDH3A
hsa03450	Non-homologous end-joining	0.007111642	MRE11/DNTT/RAD50/XRCC4/DCLRE1C/LIG4

hsa00071	Fatty acid degradation	0.007567701	ACAA1/ACSL4/ACAT1/ACADM/HADH/ACSL1/CPT2/ACOX1/ACSL6/ADH1A/ACADSB/ADH1B/ADH1C
hsa04012	ErbB signaling pathway	0.010926767	MAPK9/PIK3CB/PAK3/ARAF/ABL1/SOS2/RPS6KB1/CDKN1B/AKT3/ELK1/AKT1/CAMK2D/CAMK2G/NRG1/BRAF/TGFA/PRKCB/NRG4/STAT5B/BTC/GRB2
hsa03013	Nucleocytoplasmic transport	0.013584867	TPR/TNPO1/IPO11/KPNA3/CASC3/KPNB1/NUP98/NUP155/IPO13/GLE1/NUP153/UPF3B/XPO7/IPO8/NUP205/EEF1A1/SENP2/PHAX/XPO6/NMD3/XPOT/KPNA4/POM121/RANBP17/IPO7
hsa00620	Pyruvate metabolism	0.016538099	ME1/ACAT1/ACACB/DLD/FH/ACSS2/PDHA1/MDH2/DLAT/ADH1A/ADH1B/ADH1C/ACACA
hsa03320	PPAR signaling pathway	0.023596564	ACAA1/ME1/ACSL4/PPARD/HMGCS1/SLC27A6/ACADM/SLC27A2/PDPK1/ACSL1/DBI/CPT2/ACOX1/ACSL6/PLIN4/MMP1/GK/PLIN5
hsa00760	Nicotinate and nicotinamide metabolism	0.027043362	NT5C2/SIRT1/NUDT12/NNT/NT5C1A/SIRT5/NT5E/AOX1/NADK2/NMNAT3
hsa01212	Fatty acid metabolism	0.035817779	ACAA1/ACSL4/ACAT1/ACADM/HSD17B4/HADH/ACSL1/CPT2/ACOX1/ELOVL7/ACSL6/ACADSB/ELOVL2/ACACA
hsa00280	Valine, leucine and isoleucine degradation	0.04409474	ACAA1/HSD17B10/ACAT1/DLD/HMGCS1/ACADM/MCCC2/AOX1/HADH/HMGCLL1/ACAD8/ACADSB
hsa00230	Purine metabolism	0.046901192	ENPP4/RRM2B/GUCY2C/NT5C2/PGM1/PDE6C/IMPDH1/HDDC2/NT5C1A/GDA/PDE11A/PDE6A/AMPD3/NT5E/AK7/PRPS1/AK9/PDE6D/AK4/GMPS/AK8/HPRT1/ADPRM/ADCY5/ENTPD5/ENTPD4

Supplementary Table 3. Primers used for qRT-PCR

Name		Sequence (5’-3’)
GAPDH	Forward	TGACCTCAACTACATGGTCTACA
	Reverse	CTTCCCATTCTCGGCCTTG
JAK2	Forward	CTTGTGGTATTACGCCTGTGT
	Reverse	TGCCTGGTTGACTCGTCTATG
STAT5B	Forward	CACAGTGGATCGAAAGCCAAG
	Reverse	AGCTGGGTGGCCTTAATGTTC
